# Synchronous Neural Oscillation Between the Right Inferior Fronto-Parietal Cortices Contributes to Body Awareness

**DOI:** 10.3389/fnhum.2019.00330

**Published:** 2019-09-24

**Authors:** Naoyuki Takeuchi, Tamami Sudo, Yutaka Oouchida, Takayuki Mori, Shin-Ichi Izumi

**Affiliations:** ^1^Department of Physical Therapy, Akita University Graduate School of Health Sciences, Akita, Japan; ^2^Department of Physical Medicine and Rehabilitation, Tohoku University Graduate School of Biomedical Engineering, Sendai, Japan; ^3^Department of Education, Osaka Kyoiku University, Kashiwara, Japan; ^4^Department of Physical Medicine and Rehabilitation, Tohoku University Graduate School of Medicine, Sendai, Japan

**Keywords:** body schema, illusion, muscle spindle, transcranial alternating current stimulation, fronto-parietal cortices

## Abstract

The right inferior fronto-parietal network monitors the current status of the musculoskeletal system and builds-up and updates our postural model. The kinesthetic illusion induced by tendon vibration has been utilized in experiments on the modulation of body awareness. The right inferior fronto-parietal cortices activate during the kinesthetic illusion. We aimed to determine the relationship between the right inferior fronto-parietal cortices and body awareness by applying transcranial alternating current stimulation (tACS) to exogenously modulate oscillatory neural activity in the right fronto-parietal cortices during the kinesthetic illusion. Sixteen young adults participated in this study. We counterbalanced the order in which participants received the three types of tACS (55 Hz enveloped by 6 Hz; synchronous, desynchronous, and sham) across the subjects. The illusory movement perception induced by tendon vibration of the left extensor carpi ulnaris muscle was assessed before and during tACS. Application of synchronous tACS over the right inferior fronto-parietal cortices significantly increased kinesthetic illusion compared with sham tACS. The kinesthetic illusion during desynchronous tACS decreased from baseline. There was no change in vibration sensation during any tACS condition. The modulation of oscillatory brain activity between the right fronto-parietal cortices alters the illusory movement perception without altering actual vibration sensation. tACS over the right inferior fronto-parietal cortices is considered to modulate the neural processing involved in updating the postural model when the stimulated muscle spindle sends kinesthetic signals. This is the first study that reveals that rhythmic communication between the right inferior fronto-parietal cortices has a causal role in body awareness.

## Introduction

Somatic perception of limb position and movement of self body-parts depends on the central processing of proprioceptive information originating from the receptors in the skin, muscles, and joints (Tsakiris, 2010; Ionta et al., 2011; Blanke, 2012). Although proprioceptive signals are derived from multiple mechanoreceptor types within the body, it is known that muscle spindle receptors provide an important source of kinesthetic information (Edin and Vallbo, 1990; Ribot-Ciscar and Roll, 1998). The notion is strongly supported by experiments involving the stimulation of muscle spindle afferents using tendon vibration. Muscle spindle activity normally depends on the velocity of limb movement. However, it can also react in response to vibration stimuli (Burke et al., 1976; Roll and Vedel, 1982) where muscle afferent fibers activated by vibration send the signals as kinesthetic information to the brain causing an illusory movement perception in the absence of actual movement (Goodwin et al., 1972; Roll and Vedel, 1982; Roll et al., 1989).

Several neuroimaging studies using functional magnetic resonance imaging (fMRI) have reported that the right inferior frontal and parietal cortices are activated when experiencing kinesthetic illusion induced by tendon vibration (Cignetti et al., 2014; Amemiya and Naito, 2016; Naito et al., 2017). The right inferior fronto-parietal cortices are probably connected by the inferior branch of the superior longitudinal fasciculus tract (SLF) III (Thiebaut De Schotten et al., 2011, 2012; Rojkova et al., 2016). The right inferior fronto-parietal SLF III network has several functions, specifically, monitoring the current status of the musculoskeletal system and building-up and updating our body schema, which could be the basis for body awareness (Cignetti et al., 2014; Amemiya and Naito, 2016). Therefore, it has been speculated that this network, which underpins elements of one’s own body movement, might be essential for experiences of illusory movement perception induced by tendon vibration. However, a direct causal relationship between fronto-parietal cortices and kinesthetic illusion has not been determined and is still unclear whether the activity in fronto-parietal cortices observed by fMRI is relevant in kinesthetic illusion.

Recently, transcranial alternating electrical stimulation (tACS) has attracted attention as a non-invasive brain stimulation technique that could synchronize neural oscillation between distant brain regions by entraining brain oscillations (Herrmann et al., 2013; Vosskuhl et al., 2018; Cabral-Calderin and Wilke, 2019). Several studies have demonstrated that oscillatory synchronization of neural activity on multiple temporal scales across distant brain areas constitutes a key mechanism for cognitive and perceptive processing in humans (Struber et al., 2014; Polania et al., 2015; Violante et al., 2017). The increased rhythmic, in-phase synchrony across a network induced by synchronous tACS is thought to improve information processing by strengthening network efficiency, an effect particularly important during demanding cognitive and perceptive activity (Violante et al., 2017). In contrast, the oscillatory desynchronization between distant brain areas induced by desynchronous tACS results in deterioration of cognitive and perceptive performance (Struber et al., 2014; Polania et al., 2015). Thus, cognitive and other brain functions that involve synchronous oscillations between distant brains can be investigated using tACS.

In this study, we investigated whether synchronous and/or desynchronous tACS over the right inferior fronto-parietal cortices alters the kinesthetic illusion induced by tendon vibration. We hypothesized that an increase in kinesthetic illusion after synchronous tACS over these cortices would indicate that the synchronous neural oscillation in the fronto-parietal cortices is involved in illusory perception and in the build-up and updating of our postural model. In contrast, an increase in illusory movement perception after desynchronous tACS would indicate that the synchronous neural oscillation in the fronto-parietal cortices inhibits and/or controls illusory movement perception. The purpose of this study was to elucidate a direct causal relationship between the right inferior fronto-parietal cortices and body awareness.

## Materials and Methods

### Subjects

Sixteen young adults (7 men and 9 women, mean age 22.3 ± 2.1 years, range 20–26 years) participated in this study. They were all right-handed and had no neurological abnormalities. All participants provided written informed consent, and the protocol used in this study was approved by the local ethics committee (reference no. 2017-1-628).

### Experimental Flow

**FIGURE 1:**
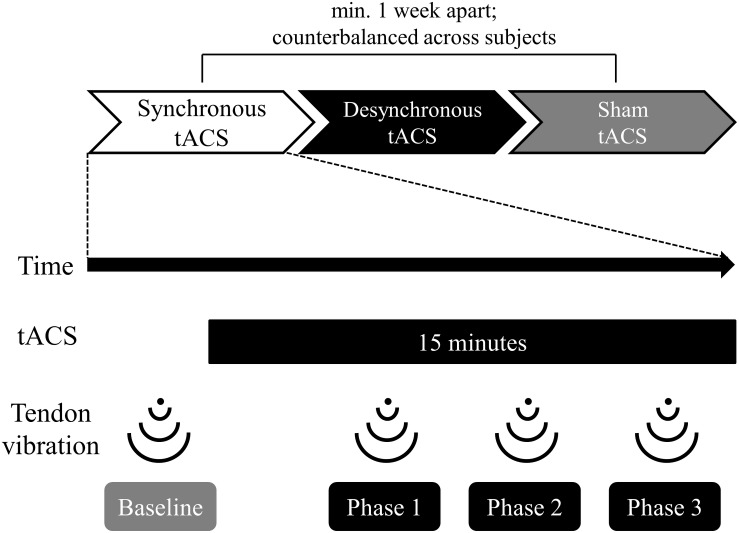
Time course of the experiment. The order in which participants received the three types of tACS (synchronous, desynchronous, and sham) was counterbalanced across subjects. Each type of tACS was separated by more than a week to avoid carryover effects. The stimulus duration for tACS was 15 min. Participants received tendon vibration before tACS (baseline), 5 min after the start of tACS (phase 1), 9 min after the start of tACS (phase 2), and 13 min after the start of tACS (phase 3). Participants reported vibration perception and kinesthetic illusion using a self-assessment scale immediately after vibration at each phase.

Figure 1 shows the experimental flow. We counterbalanced the order in which the participants received the three types of tACS (synchronous, desynchronous, and sham). Administration of each type of tACS was separated by more than 1 week to avoid carryover effects. The illusory movement perception induced by tendon vibration was assessed once before tACS and three times during tACS.

### Vibration Stimulation

We induced vibration stimulation using a vibration machine (around 110 Hz; Thrive MD-01, Daito Ltd., Osaka, Japan) that had a small custom-made contact surface (approximately 0.8 cm^2^) at the tip. We vibrated the tendon of the left extensor carpi ulnaris muscle for 20 s to elicit an illusory wrist flexion. During the tendon vibration, the participants closed their eyes and relaxed their limbs without making unnecessary movements. During each vibration session, both the left and right arm of each participant was placed on the desk in a straight position. We confirmed that there was no visible wrist movement during tendon vibration.

### Application of tACS

**FIGURE 2:**
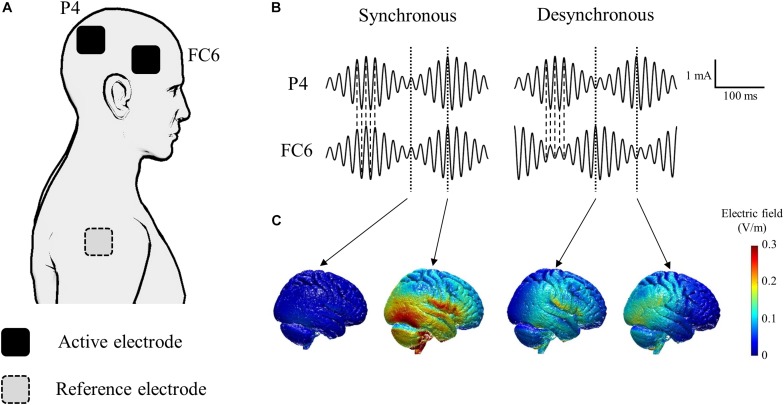
Schematic of tACS applied to each of the active electrodes. **(A)** Active electrodes were placed on the scalp over the right inferior frontal gyrus (FC6) and the right inferior parietal lobule (P4). The reference electrode was placed on the right shoulder. **(B)** The tACS waveform consisted of oscillatory currents at 55 Hz modulated by a 6 Hz envelope. Synchronous tACS is an in-phase stimulation aligned at 0° while desynchronous tACS is an anti-phase stimulation shifted by 180°. **(C)** Electric field simulation. We used ROAST, a fully automated toolbox for realistic current flow models of human (Huang et al., 2019).

The tACS was delivered through gel-sponge electrodes (surface area, 25 cm^2^; IOGEL; IOMED, United States) from two battery-driven constant current stimulators (DC-Stimulator Plus, NeuroConn GmbH, Germany) connected to a common reference. We placed active electrodes over FC6 and P4 according to the international electroencephalography (EEG) 10-20 system to stimulate the right inferior frontal gyrus and the right inferior parietal lobule (Hogeveen et al., 2015; Cai et al., 2016; Figure 2A). The reference electrode was placed on the right shoulder. We induced oscillatory currents at 55 Hz that were modulated by a 6 Hz envelope to closely mimic the endogenous phase-amplitude modulation phenomenon occurring in the human cortex during cognitive tasks (Canolty et al., 2006; Polania et al., 2015). It is hypothesized that the coupling between theta and gamma brain rhythms coordinates activity in different cortical areas, thus providing a mechanism for effective communication during cognitive processing in humans (Canolty et al., 2006). Synchronous tACS is an in-phase stimulation aligned at 0 degrees while desynchronous tACS is an anti-phase stimulation shifted by 180 degrees (Figure 2B). The maximum peak-to-peak current stimulation was 2 mA. During tACS, the current was ramped up for 30 s, followed by 15 min stimulation, and then it was ramped down for 30 s. During sham stimulation, the current was ramped up for 30 s, followed by 30 s stimulation, and then it was ramped down for 30 s to mimic the real tACS. The tACS waveform was controlled by an external controller (DC-StimEditor, Medical Try System, Japan). We used ROAST, a toolbox for realistic current-flow models to show how the tACS stimulated the cortex in this study (Figure 2C; Huang et al., 2019). MNI-152 standard head was used for a finite element head model (Grabner et al., 2006). The position of active electrodes for the simulation model was FC6 and P4, same as this study, but that of reference electrode was right neck, near the right shoulder because ROAST uses the segmentation algorithm that applies it to the head and neck without the shoulder (Huang et al., 2019).

### Self-Assessment Parameters

In order to evaluate the illusory movement perception, participants were asked to report their own illusory perceptions related to persistence, vividness, and strength using a 100-point visual analog scale (0 points: not at all, 100 points: completely) (Naito et al., 1999). Participants also reported their own perception about vibration intensity (vibration perception score) using a 100-point visual analog scale to evaluate the habituation from the vibration. Subjects reported these self-assessment parameters immediately after vibration at each phase (baseline; before tACS, phase 1; 5 min after the start of tACS, phase 2; 9 min after the start of tACS, and phase 3; 13 min after the start of tACS).

### Statistical Analysis

A two-way repeated-measures analysis of variance (ANOVA) was used to determine the effects of stimulation (synchronous, desynchronous, and sham) and period (baseline, phase 1, phase 2, and phase 3) on the self-assessment parameters. All statistical analyses were performed with SPSS version 24.0 (SPSS Inc., Chicago, IL, United States). The alpha was set at 0.05 for significance and a *post hoc* analysis was performed using Bonferroni’s correction. All data were normalized by conversion to percentage changes from the baseline values.

## Results

Participants did not report any adverse side effects (headache, intolerable pain, nausea, skin lesions, etc.) during the study. Fifteen of the 16 participants reported experiencing vivid movement perception of left wrist flexion during tendon vibration. We excluded one participant who reported experiencing no kinesthetic illusion from our study.

### Self-Assessment Parameters

**FIGURE 3:**
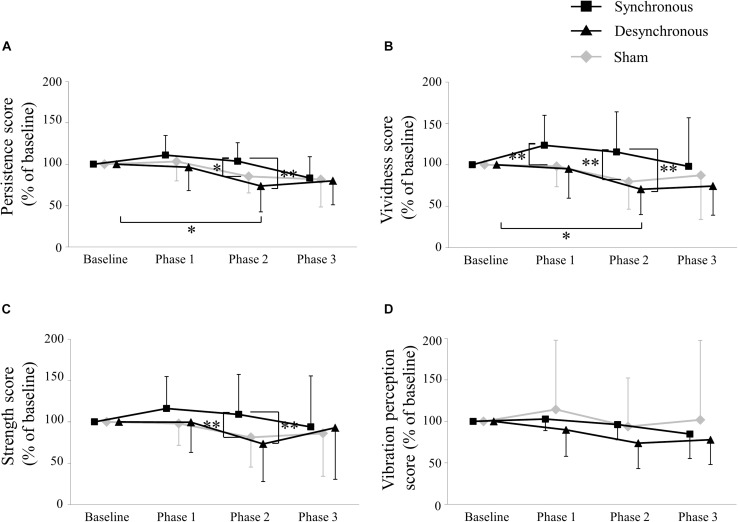
Self-assessment score. **(A)** Persistence score. The persistence score during synchronous tACS was larger than during sham (*p* = 0.033) and during desynchronous tACS (*p* = 0.0002) at Phase 2. During desynchronous tACS, the persistence score at Phase 2 was lower than at baseline (*p* = 0.033). **(B)** Vividness score. The vividness score during synchronous tACS was larger than during sham (*p* = 0.003) at Phase 1, and during sham (*p* = 0.004) and during desynchronous tACS (*p* = 0.007) at Phase 2. During desynchronous tACS, the vividness score at Phase 2 was lower than at baseline (*p* = 0.013). **(C)** Strength score. The strength score during synchronous tACS was larger than during sham (*p* = 0.003) and during desynchronous tACS (*p* = 0.004) at Phase 2. **(D)** Vibration perception score. ^∗^*p* < 0.05, ^∗∗^*p* < 0.01, error bar: standard deviation.

Figure 3 depicts the self-assessment parameters. A two-way repeated-measures ANOVA of the persistence score showed a significant effect of stimulation [*F*(_2_, _28_) = 5.038, *p* = 0.014], period [*F*(_3_, _42_) = 6.732, *p* = 0.0008] and an interaction between them [*F*(_6_, _84_) = 3.387, *p* = 0.005]. *Post hoc* testing revealed that the persistence score during synchronous tACS was larger than during sham (*p* = 0.033) and during desynchronous tACS (*p* = 0.0002) at Phase 2. During desynchronous tACS, the persistence score at Phase 2 was lower than at baseline (*p* = 0.033).

A two-way repeated-measures ANOVA of the vividness score showed a significant effect of stimulation [*F*(_2_, _28_) = 7.803, *p* = 0.002], period [*F*(_3_, _42_) = 2.931, *p* = 0.044] and an interaction between them [*F*(_6_, _84_) = 3.831, *p* = 0.002]. *Post hoc* testing revealed that the vividness score during synchronous tACS was larger than during sham (*p* = 0.003) at Phase 1, and during sham (*p* = 0.004) and during desynchronous tACS (*p* = 0.007) at Phase 2. During desynchronous tACS, the vividness score at Phase 2 was lower than at baseline (*p* = 0.013).

A two-way repeated-measures ANOVA of the strength score showed a significant effect of stimulation [*F*(_2_, _28_) = 4.284, *p* = 0.024] and an interaction between stimulation and period [*F*(_6_, _84_ = 3.335, *p* = 0.005] but no period [*F*(_3_, _42_) = 1.462, *p* = 0.239]. *Post hoc* testing revealed that the strength score during synchronous tACS was larger than during sham (*p* = 0.003) and during desynchronous tACS (*p* = 0.004) at Phase 2.

A two-way repeated-measures ANOVA of the vibration perception score showed no significant effect of stimulation [*F*(_2_, _28_ = 1.005, *p* = 0.379] and period [*F*(_3_, _42_) = 2.365, *p* = 0.085] or an interaction between them [*F*(_6_, _84_) = 1.001, *p* = 0.430]. Self-assessment scores in each individual participant are shown in Table 1 and box plots of self-assessment scores are shown in Supplementary Figure 1.

**TABLE 1:** Self-assessment score in each individual participant.

**Subject**	**Synchronous**				**Desynchronous**				**Sham**			
			
**No**	**Baseline**	**Phase 1**	**Phase 2**	**Phase 3**	**Baseline**	**Phase 1**	**Phase 2**	**Phase 3**	**Baseline**	**Phase 1**	**Phase 2**	**Phase 3**
**PERSISTENCE SCORE**												
1	57	71	72	35	53	59	48	55	74	84	62	66
2	55	52	49	35	65	45	26	28	37	37	21	11
3	55	84	64	42	49	52	15	53	82	98	61	27
4	89	91	45	34	70	58	16	35	57	61	54	71
5	73	90	56	35	99	91	42	50	78	86	32	42
6	88	69	68	41	81	23	33	8	76	67	49	20
7	71	77	83	74	83	97	99	89	95	48	99	82
8	77	72	88	74	90	82	86	74	86	85	78	76
9	63	67	65	70	62	64	42	55	90	69	74	84
10	91	100	99	99	100	100	100	100	100	100	100	100
11	47	62	61	38	52	81	55	48	56	89	48	34
12	89	89	91	91	89	87	92	94	74	87	90	90
13	99	73	99	100	93	81	68	72	68	71	63	81
14	51	80	67	57	72	90	68	69	69	64	65	75
15	82	89	92	82	93	73	73	79	81	90	75	73
Mean	72.5	77.7	73.3	60.5	76.7	72.2	57.5	60.6	74.9	75.7	64.7	62.1
SD	16.9	12.9	17.6	25.0	17.6	21.6	28.9	25.5	16.2	18.2	22.5	27.8

**VIVIDNESS SCORE**												

1	65	73	74	46	59	61	69	63	72	80	65	65
2	50	55	46	39	72	61	51	28	64	46	24	15
3	69	84	56	39	61	52	16	39	90	87	61	26
4	88	91	45	33	79	59	15	48	86	62	44	60
5	59	80	43	24	83	80	32	35	79	86	32	13
6	83	59	64	51	51	11	22	6	78	46	28	16
7	42	67	59	82	97	100	99	100	94	85	99	96
8	80	75	87	73	85	76	78	74	84	84	76	71
9	57	64	55	62	42	60	39	56	72	64	68	80
10	33	64	74	86	33	28	33	45	23	33	38	44
11	44	63	52	45	41	70	42	34	14	12	11	10
12	75	78	83	72	78	68	54	54	57	58	54	57
13	99	72	87	67	93	93	68	65	90	71	63	75
14	61	81	87	67	76	99	50	53	52	63	53	72
15	15	28	32	14	45	23	20	17	30	43	21	53
Mean	61.3	68.9	62.9	53.3	66.3	62.7	45.9	47.8	65.7	61.3	49.1	50.2
SD	22.3	15.1	18.1	21.4	20.4	26.4	24.6	23.3	25.6	22.1	23.8	27.9

**STRENGTH SCORE**												

1	73	63	73	45	57	55	54	57	83	89	57	59
2	49	54	36	32	77	57	44	35	68	52	22	17
3	70	92	87	53	80	82	15	80	100	99	80	26
4	89	92	46	32	79	59	14	49	88	62	42	58
5	58	80	37	16	82	79	31	50	81	85	31	12
6	79	58	55	42	52	9	14	6	75	41	26	16
7	36	75	73	76	71	99	100	93	79	65	97	92
8	83	76	87	73	87	68	78	81	79	87	73	72
9	57	65	54	58	39	59	34	51	70	60	63	75
10	36	66	79	93	18	29	33	52	24	36	39	45
11	43	52	39	41	70	81	45	43	47	51	42	53
12	84	74	87	71	83	76	55	61	63	67	73	67
13	98	73	86	67	95	87	68	73	91	65	56	82
14	61	81	95	53	76	99	33	53	34	50	33	59
15	83	72	77	79	80	58	80	71	85	82	73	70
Mean	66.6	71.5	67.4	55.4	69.7	66.5	46.5	57.0	71.1	66.1	53.8	53.5
SD	19.8	12.1	20.8	21.1	20.3	24.4	26.0	21.3	21.3	18.8	22.3	25.2

**VIBRATION PERCEPTION SCORE**												

1	85	72	80	67	68	68	70	71	75	88	67	67
2	65	69	66	64	79	63	49	56	72	68	55	67
3	80	92	84	36	91	51	34	51	73	86	79	52
4	89	84	67	42	81	58	19	44	91	85	56	66
5	48	49	34	8	83	51	33	33	82	94	33	13
6	66	53	63	55	91	11	40	7	78	41	12	13
7	67	75	54	92	65	94	65	66	93	58	82	72
8	84	86	90	72	84	80	86	86	85	87	78	78
9	68	61	73	68	52	62	54	56	75	65	67	75
10	90	94	94	99	100	100	100	100	100	99	100	98
11	56	76	36	59	77	70	71	58	91	86	79	75
12	81	81	87	74	86	78	61	63	61	66	70	70
13	100	99	100	100	97	87	62	84	94	80	63	84
14	66	79	92	52	77	100	35	54	12	49	34	52
15	85	83	77	79	75	80	89	88	79	62	75	65
Mean	75.3	76.9	73.1	64.5	80.4	70.2	57.9	61.1	77.4	74.3	63.3	63.1
SD	14.3	14.4	20.1	24.5	12.5	23	23.2	23.5	20.9	17.2	22.6	23.3

## Discussion

In this study, we investigated whether illusory movement perception is altered by tACS over the right inferior fronto-parietal cortices that monitor the current status of the musculoskeletal system and build-up and update our postural model. Our results demonstrated that transcranially inducing oscillatory modulation between the right inferior frontal and parietal cortices affects kinesthetic illusion without a change in the actual vibration sensation. To the best of our knowledge, this is the first study that elucidates the causal role of the synchronous neural oscillation between the right inferior fronto-parietal cortices on body awareness.

### The Right Fronto-Parietal SLF III Network Is Essential for Kinesthetic Illusion

For one to perceive postural change during illusion, the brain has to build-up and update the postural model by monitoring the current status of the musculoskeletal system. The fronto-parietal SLF III network, which connects a broad range of inferior fronto-parietal regions, appears to be involved in the neuronal processes involved in body schema (Cignetti et al., 2014; Amemiya and Naito, 2016). We targeted the right hemisphere since the right SLF III network has a greater volume than the left SLF III network (Thiebaut De Schotten et al., 2011). This could be explained by several factors including greater fiber myelination, more axons, and larger axonal diameter in the right SLF III network (Thiebaut De Schotten et al., 2011). The greater tract volume might also be suitable for the speedy processing of massive amounts of complex information derived from our body (Naito et al., 2017). Indeed, right-side dominant activity in the inferior frontal and parietal cortices has been repeatedly confirmed in participants experiencing kinesthetic illusion regardless of the vibration site (Naito et al., 2005; Amemiya and Naito, 2016). Thus, it is assumed that kinesthetic illusion activates the right fronto-parietal cortices, however, there has been no direct evidence that the synchronous neural oscillation between the right fronto-parietal cortices causes the kinesthetic illusion. Our study demonstrated that synchronous tACS over the right inferior fronto-parietal cortices significantly increased kinesthetic illusion compared to desynchronous and sham tACS. Increased rhythmic synchrony across a network is thought to improve cognitive and perceptive processing by strengthening network efficiency (Violante et al., 2017). Therefore, synchronous tACS over the right fronto-parietal cortices might strength the awareness of postural change during an illusion by facilitating neural processes involved in building-up and updating the postural model of our limbs when the stimulated muscle spindle sends kinesthetic signals. In contrast, kinesthetic illusion reduced during desynchronous tACS compared with before stimulation. These results indicate that the oscillatory desynchronization between the fronto-parietal cortices during desynchronous tACS deteriorated the neural processing involved in building-up and updating the postural model.

The Global Neuronal Workspace (GNW) model of conscious processing also suggests that communication between the right fronto-parietal cortices is essential for illusory movement perception (Dehaene and Changeux, 2011). This model proposes that conscious processing occurs when stimulus information is propagated to different brain areas through a network of neurons with long-range axons densely distributed in frontal and parietal cortices. Moreover, the GNW model proposes that conscious processing requires cortico-cortical synchronization at gamma frequencies such as those used for tACS in this study (Dehaene and Changeux, 2011).

Unlike synchronous tACS, desynchronous tACS did not significantly alter the kinesthetic illusion compared with the sham condition. This is possible because of the neural habituation caused by repeated tendon vibration. The neural habituation might induce changes in the kinesthetic illusion depending on the passage of sessions; therefore, it might have influenced the outcome of significant effects of the period in the persistence and vividness scores. In the sham group, the illusionary movement perception decreased during the tACS session, although not significantly. Based on this, it might be difficult to detect differences in kinesthetic illusion between desynchronous and sham tACS. As another reason, it is possible that the desynchronous tACS parameters in this study might be insufficient to impede the inferior fronto-parietal network related to the kinesthetic illusion. The synchronous and desynchronous tACS conditions differ in terms of peak field strength and focality. This is because the current strength at the reference electrode is twice the current strength at the active electrode in synchronous condition, but the current strength at the reference electrode is reduced by the opposing phases of two active electrodes in desynchronous condition (Violante et al., 2017). Therefore, the desynchronous stimulation might be inappropriate as a control condition for the synchronous stimulation (Saturnino et al., 2017). We might not have used the optimal desynchronous tACS expected to alter the kinesthetic, given that studies on lesions in the human brain have demonstrated that damage in the inferior frontal cortex and parietal cortex impairs own-body perception (Berlucchi and Aglioti, 1997; Berti et al., 2005).

We observed that the effect of tACS on the kinesthetic illusion changed with the duration of tACS. The change in kinesthetic illusion at Phase 1 could only be detected in the vividness score. This could be because the duration of tACS at Phase 1 was still short, and thus could not alter all the subscores of kinesthetic illusion. However, there was no significant difference in the kinesthetic illusion between the three tACS groups at Phase 3, which was considered to have sufficient duration of stimulation. As a possible mechanism, the neural habituation with repeated tendon vibration might be stronger at Phase 3, which was the last session; therefore, making it difficult to detect differences in the kinesthetic illusion between the tACS groups.

### Possibility That Other Cortical Areas Contribute to the Kinesthetic Illusion

Besides the fronto-parietal cortices, previous studies have reported that the motor areas and higher-order somatosensory areas, which work together with the fronto-parietal network in forming body perception, are activated during kinesthetic illusion (Naito et al., 1999; Naito et al., 2005; Amemiya and Naito, 2016). This illusion is basically a bottom-up sensory event where motor intention and voluntary generation of motor commands are not particularly required. The right inferior fronto-parietal cortices recognize the postural change of one’s body based on bottom-up sensory afferent input. Oscillatory synchronization between the right inferior frontal and parietal cortices selectively affects kinesthetic illusion but not somatic sensation, which is the vibration sensation processed primarily in the somatosensory area. Considering these findings, the right inferior frontal-parietal network might play an essential role in higher-order perceptive processing of illusory movement perception, although the motor and higher-order somatosensory areas might also be involved. Anatomical findings support this notion, because the right inferior-parietal SLF III network likely communicates with the motor areas through the frontal aslant tract and with neighboring higher-order somatosensory areas (Naito et al., 2016; Rojkova et al., 2016). However, future studies should evaluate the alteration of kinesthetic illusion after brain stimulation over these areas to determine the site primarily involved in the kinesthetic illusion. Moreover, additional research should evaluate whether tACS over the right fronto-parietal cortices affects the illusionary movement sensation in the right (ipsilateral) hand during tendon vibration to confirm the right dominance of the fronto-parietal network activity in kinesthetic illusion (Cignetti et al., 2014; Amemiya and Naito, 2016). This approach would also determine whether our findings resulted from the effect of tACS over the motor and higher-order somatosensory networks on the vibrated contralateral limb.

### Limitations

There were several limitations to this study. First, we did not evaluate the physiological brain activity to investigate the mechanism of the effect of tACS, although we hypothesized that synchronous tACS over the frontal and parietal cortices enhances synchronous neural oscillation between the two stimulated brain regions. Studies that simultaneously combine tACS with EEG or fMRI could explain this entrainment hypothesis. Second, we only compared synchronous tACS over the frontal and parietal cortices with desynchronous and sham stimulation. Since synchronous tACS and desynchronous tACS resulted in opposite effects on the kinesthetic illusion, it is unlikely that our results might be due to stimulation of either brain region alone. However, to conclude that our results were due to synchronous stimulation of these two regions, the same oscillatory tACS over the frontal and parietal cortex separately should serve as a control condition. Third, we measured only the kinesthetic illusion and vibration perception as indicators of tACS effect over the right fronto-parietal cortices. However, it is known that right fronto-parietal cortices are involved in the attention and egocentric space representation (Corbetta and Shulman, 2002; Thiebaut De Schotten et al., 2011; Saj et al., 2014). Therefore, future studies must evaluate the attention and egocentric space representation during tACS to assess the possibility that the changes in these functions might affect the kinesthetic illusion during tACS. Forth, the electrical perception of the extracephalic reference electrode on the right shoulder might have influenced the kinesthetic illusion on the left wrist as somatic stimulation despite being on the contralateral side. Applying a tACS protocol with all electrode positions located on the head could reduce the possibility of electrical perception of the reference electrode influencing body awareness. Finally, although our tACS protocol was informed by previous neuroimaging studies on kinesthetic illusion and tACS studies on cognitive processing (Cignetti et al., 2014; Polania et al., 2015; Amemiya and Naito, 2016), it is important to determine the optimal parameters for inducing kinesthetic illusion, such as stimulation intensity, frequency, and electrode positioning. Although we did not use brain MRI data from the actual participants, the electric field simulation indicated that our protocol might stimulate the temporal cortex more than the parietal cortex (Figure 2C). Therefore, future studies should determine the optimal tACS parameters using different tACS frequencies or/and using ring electrodes to stimulate more focalized cortical areas.

## Conclusion

Transcranially inducing modulation of oscillatory brain activity between the right inferior fronto-parietal cortices selectively affected illusory movement perception without altering the actual vibration sensation. Our findings demonstrate the presence of a direct link between kinesthetic illusion and large-scale synchronization across the right fronto-parietal network. These results support the notion that synchronous oscillatory activity in large-scale neuronal networks is an essential mechanism for conscious perception and cognition.

## Data Availability Statement

The datasets generated for this study are available on request to the corresponding author.

## Ethics Statement

The studies involving human participants were reviewed and approved by the local ethics committee of the Tohoku University Graduate School of Medicine. The participants provided their written informed consent to participate in this study.

## Author Contributions

NT was the lead writer of this manuscript and was responsible for designing the study, acquisition of data, analysis, and interpretation of data. TS and YO participated in acquisition and analysis of data. TM participated in the analysis and interpretation of data. NT and S-II assisted in data interpretation and contributed to the writing of the manuscript. All authors have read and approved the final manuscript.

## Conflict of Interest

The authors declare that the research was conducted in the absence of any commercial or financial relationships that could be construed as a potential conflict of interest.

## Supplementary Material

FIGURE S1 Box plot of self-assessment score. **(A)** Persistence score. **(B)** Vividness score. **(C)** Strength score. **(D)** Vibration perception score. For each box plot, the plain line within the box indicates the median, and whiskers extend from the box to the lowest and highest data points that are still within a 1.5-interquartile range of the lower and upper quartiles. Dots indicate values beyond the whisker ends. ^∗^*p* < 0.05, ^∗∗^*p* < 0.01 (asterisk without a line indicates a *p*-value comparison with baseline).
