# Supplementary figures and images for: Synchronous Neural Oscillation Between the Right Inferior Fronto-Parietal Cortices Contributes to Body Awareness
Source: Front Hum Neurosci. 2019 Sep 24;13:330. doi: 10.3389/fnhum.2019.00330 (PMC6769041; doi:10.3389/fnhum.2019.00330)

(a)

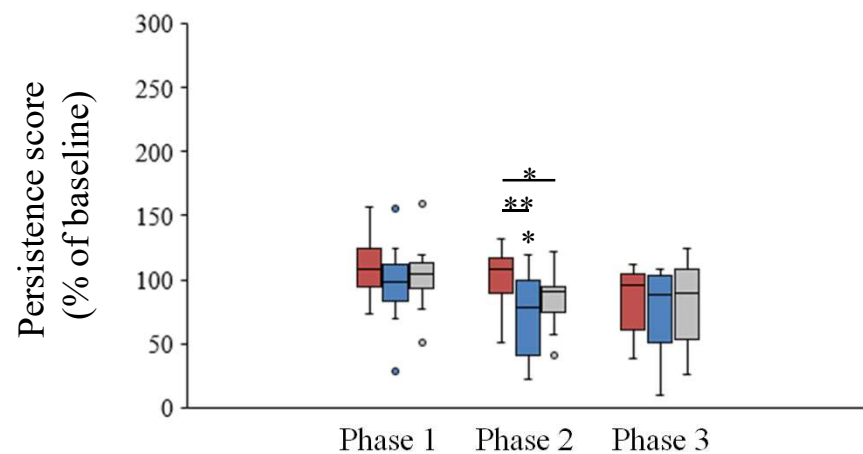

(b)

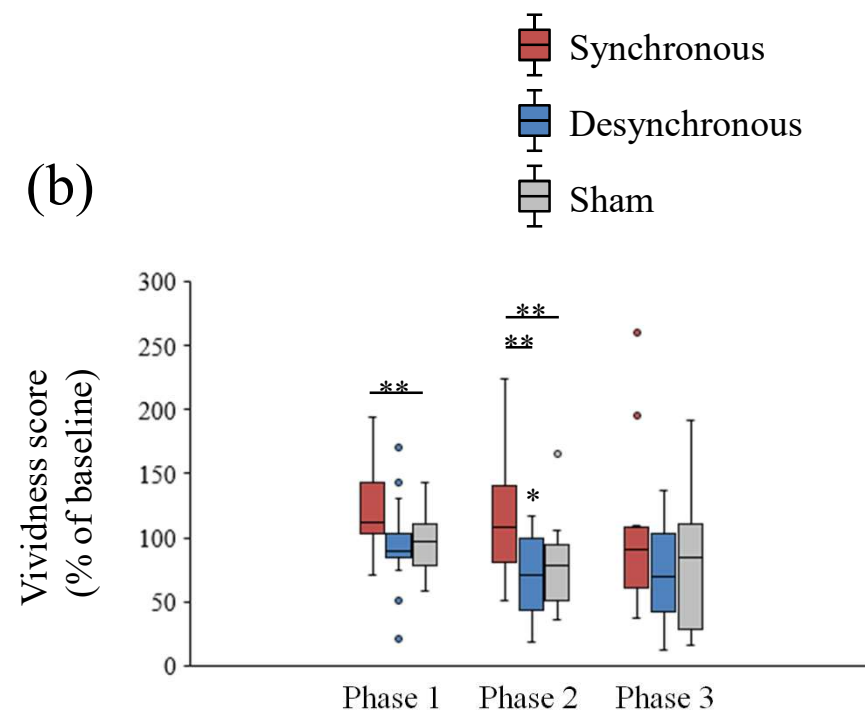

(c)

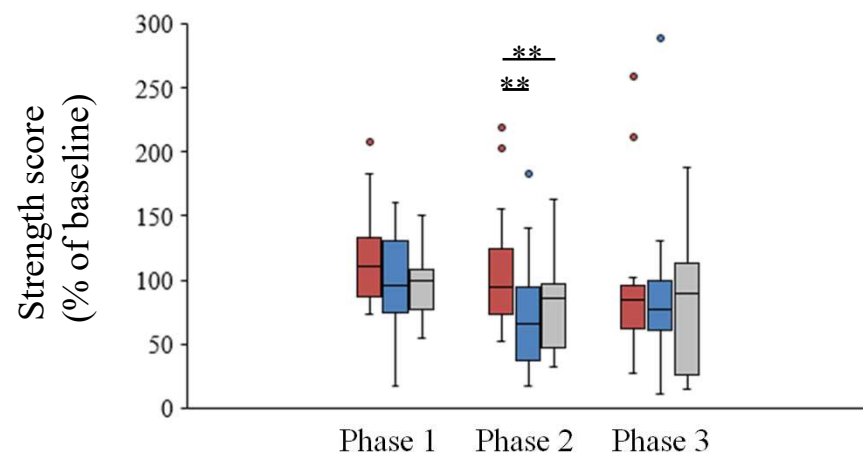

(d)

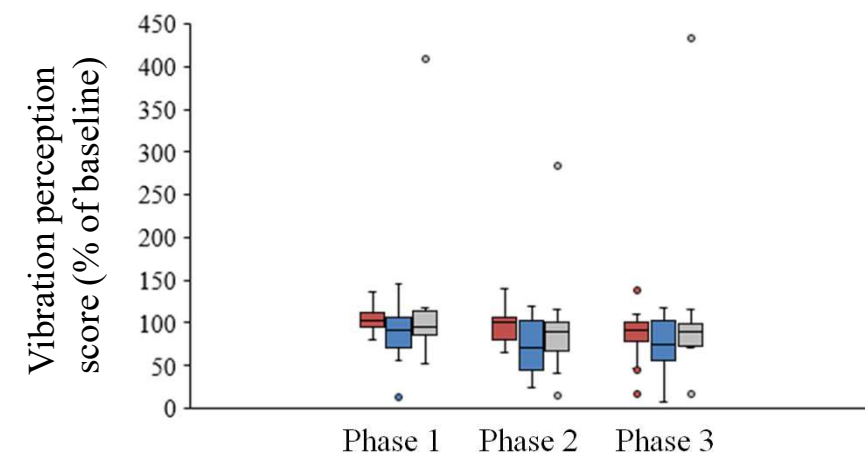

Supplement: FIGURE S1 — Box plot of self-assessment score. (A) Persistence score. (B) Vividness score. (C) Strength score. (D) Vibration perception score. For each box plot, the plain line within the box indicates the median, and whiskers extend from the box to the lowest and highest data points that are still within a 1.5-interquartile range of the lower and upper quartiles. Dots indicate values beyond the whisker ends. ∗p < 0.05, ∗∗p < 0.01 (asterisk without a line indicates a p-value comparison with baseline). [file Image_1.pdf]
